# Repeated-Measures Associations Between Countermovement Jump Variables and Driving Performance in Sub-Elite Male Golfers

**DOI:** 10.3390/sports14100439

**Published:** 2026-10-08

**Authors:** Thomas E. Bright, Matthew Ellis, Chris Bishop, Sarah Martin, Alexei Glass, Jonathan D. Hughes

**Affiliations:** 1 School of Sport, Plymouth Marjon University, Plymouth PL6 8BH, UK; mellis@marjon.ac.uk (M.E.); smartin@marjon.ac.uk (S.M.); alexeiglass@aol.com (A.G.); 2 Youth Physical Development Centre, Cardiff School of Sport and Health Sciences, Cardiff Metropolitan University, Cardiff CF23 6XD, UK; jdhughes@cardiffmet.ac.uk; 3 Tournament Golf College, St Mellion Estate, Cornwall PL12 6SD, UK; 4 London Sport Institute, Faculty of Science and Technology, Middlesex University, London NW4 4BT, UK; c.bishop@mdx.ac.uk; 5 PGA European Tour Group, Virginia Water, Surrey GU25 4LZ, UK; 6 Sports Academy, PGA of America, Frisco, TX 75034, USA

**Keywords:** jump momentum, clubhead speed, ball speed, smash factor, Bayesian quantile regression, athlete monitoring

## Abstract

Background: Countermovement jump (CMJ) force–time variables are commonly used to monitor physical qualities linked to golf driving performance, but most evidence derives from single time-point, mean-based correlations. This study examined repeated-measures associations between CMJ variables and driving performance in sub-elite male golfers using Bayesian quantile regression. Methods: Twenty-eight male golfers (handicap 0.8 ± 1.9) completed two sessions, one week apart, of maximal CMJs and driver swings. Jump height, jump momentum and peak propulsive power were calculated from the CMJs, while clubhead speed (CHS), ball speed and smash factor were obtained from the driver swings. Results: Between-session absolute reliability was excellent for all variables (CV upper 95% credible interval [CrI] ≤ 3.8%), whereas relative reliability was excellent for CHS and all CMJ variables (ICC lower 95% CrI ≥ 0.96), moderate for ball speed (lower 95% CrI = 0.71) and poor for smash factor (lower 95% CrI = 0.00). All CMJ variables showed positive associations with CHS across percentiles in both sessions, with little evidence of session-related change for jump momentum or peak propulsive power. In contrast, the jump height–CHS association weakened in session two at the median and upper percentiles (from 1.3–1.4 to 1.0–1.2 mph per 0.05 m increase in jump height; 98–99% posterior probability of a decrease). Associations between CMJ variables and ball speed and smash factor were uncertain, with 95% CrI spanning zero in almost all cases. Conclusions: Repeated-measures designs may add value in applied golf settings. Jump momentum and peak propulsive power showed comparatively stable associations with CHS across sessions, although both scale with body mass, which may partly account for this stability. They may therefore be useful complementary monitoring variables, but their predictive value and responsiveness to training are yet to be established.

## 1. Introduction

Driving distance is consistently identified as one of the most influential determinants of scoring performance in professional golf [1,2,3]. Although a player’s final score reflects contributions from many shot types, evidence suggests a hierarchy of importance: driving, followed by approach play (i.e., shots intended to reach the green), putting, and finally the short game (i.e., shots playing from within approximately 100 yards of the green) [2]. Notably, the ‘strokes gained’ analysis suggests that a 20-yard increase in driving distance translates into approximately 0.75 strokes saved per round, or three strokes across a standard four-round event, which is enough to meaningfully impact both rankings and prize money [2]. Owing to several variables affecting driving distance (e.g., centredness of strike, ball temperature, launch conditions, club design and environmental factors) [4,5,6,7], researchers have mostly employed clubhead speed (CHS) as a performance outcome [4,8]. However, while CHS is a key determinant of driving distance, ball speed represents a more direct outcome of club–ball impact and is more closely associated with subsequent ball–flight characteristics [4], and smash factor (i.e., ratio of ball speed to CHS) indicates how effectively CHS is transferred to the ball. Therefore, consideration of all three variables may provide a more holistic and ecologically valid assessment of driving performance.

In accordance with Newton’s Second Law of Motion, the generation of CHS in part depends on the ground reaction forces (GRFs) produced at the foot–floor interface, as these forces drive the body’s subsequent motion [9]. Of the GRFs observed during the golf swing, the vertical component is the largest, and its magnitude is typically greater in players who achieve a higher CHS [9]. Indeed, vertical GRFs (vGRF) have been reported to exhibit a systematic pattern, decreasing during the latter backswing, reaching a minimum at the top, and increasing rapidly to a peak during the early-to-mid downswing [9,10]. A comparable reliance on vGRF production is evident in the countermovement jump (CMJ) to accelerate the body’s centre of mass into flight [11]. Given these parallels, several studies have investigated the CMJ as a practical means of assessing the force–time characteristics that may be associated with golf driving performance [12,13,14,15,16].

In a sample of category one golfers (handicap ≤ 5.4), Wells et al. [14] reported strong relationships between CHS and CMJ positive impulse, net impulse, mean power, peak power, peak vGRF and vGRF at zero velocity (*r =* 0.517 to 0.695; *p* < 0.001), despite no clear relationship with jump height (*r* = 0.176). More recently, Brennan et al. [12] compiled evidence through a meta-analysis indicating that the strongest pooled associations with CHS were observed for jump impulse (*r* = 0.68; 95% confidence interval (95% CI) = 0.56 to 0.77), with jump displacement showing a relatively weaker association (*r* = 0.49; 95% CI = 0.27 to 0.65). However, the impulse data were reported as positive impulse, which includes the bodyweight component and therefore scales strongly with body mass, whereas jump displacement is independent of body mass. The authors argued that this favours impulse- or momentum-based measures, as body mass contributes to CHS, but it also means the stronger association may partly reflect body size rather than a more informative measure of jumping ability [17]. Similarly, Johansen et al. [13] reported strong associations between driver CHS and CMJ impulse and peak power in elite male golfers (*r* = 0.78; *p* < 0.001). Collectively, these findings support the CMJ as a monitoring tool in golf; however, research has focused primarily on CHS as the outcome of interest, with ball speed and smash factor receiving comparatively less attention [4,18].

Brennan et al. [19] reported moderate to very large associations between CMJ variables and both CHS and ball speed in high-level amateur golfers (*r* = 0.41 to 0.82), with jump height showing the strongest associations overall (*r* = 0.73 and 0.82, respectively), whereas smash factor showed the weakest and most variable associations (*r* = −0.11 to 0.69). However, jump height may not fully capture the relationship, as a heavier golfer may achieve a higher CHS despite a modest jump height [18]. By contrast, Robinson et al. [20] reported a trivial negative association between jump height and CHS in elite female amateur golfers (*r* = −0.11), despite trivial-to-large associations between other CMJ variables and ball speed (*r* = 0.05 to 0.62). Associations therefore vary considerably between studies, and not all of the studies pooled in the meta-analysis by Brennan et al. [12] reported clear relationships, with samples spanning male and female golfers of varying skill levels. As such, while CMJ variables may contribute to CHS, their influence on ball speed and smash factor appears more variable and less predictable. Most existing associations are also based on single time-point observations [21,22], so whether they remain stable across repeated sessions is unclear. Together, this supports approaches that quantify uncertainty directly and examine whether associations hold across the performance distribution and across sessions.

Therefore, the aim of this study was to examine associations between CMJ force–time variables and driver CHS, ball speed and smash factor across the performance distribution (25th, 50th, 75th and 90th percentiles) in young adult male sub-elite golfers, using a repeated-measures Bayesian quantile regression approach. In doing so, this study sought to determine whether these associations varied across percentiles and remained consistent across two testing sessions.

## 2. Materials and Methods

### 2.1. Participants

Following simulations, the study aimed to recruit thirty-five male golfers who participate as part of their routine annual performance testing. The final sample size (*n* = 28; age: 19.9 ± 1.0 years; stature: 1.80 ± 0.06 m; body mass: 76.6 ± 13.9 kg; handicap: 0.8 ± 1.9) reflected the available cohort but was formally re-evaluated using a simulation-based Bayesian approach (detailed in ‘Statistical Analysis’ section). Participants were defined as sub-elite according to the International Golf Federation consensus statement (“PGA teaching professionals, amateurs competing in regional/county/state tournaments, or with handicap ≤ 5”) [23] and all were free from musculoskeletal injury. All participants provided written informed consent after being made aware of the study’s aims, benefits and potential risks. Ethical approval was granted by the University Research Ethics Panel (protocol code EP241; approved 14 January 2025).

### 2.2. Testing Procedures

All data were collected in a private golf swing studio. Participants attended two testing sessions separated by one week, each at the same time of day, with both following an identical protocol. Between sessions, participants were asked to avoid any high-intensity activity or anything likely to cause soreness or fatigue (e.g., high-intensity gym sessions or unaccustomed exercise), although they were permitted to continue playing golf. In session one, standing height was measured to the nearest 0.01 m using a portable stadiometer (SECA 213, Vogel & Halke, Hamburg, Germany) and body mass was recorded to the nearest 0.1 kg using digital scales (SECA 875, Vogel & Halke, Hamburg, Germany). Participants were then guided through a standardised warm-up consisting of 10 repetitions each of body weight squats, reverse lunges, bilateral and unilateral glute bridges and press ups. Participants were permitted five minutes of rest prior to completing driving assessments.

#### 2.2.1. Clubhead Speed, Ball Speed and Smash Factor

Prior to the driving assessments, participants completed five preparatory swings with their driver, progressively increasing effort from submaximal to maximal based on perceived intensity. Participants used their own custom-fitted driver and set up their own tee for each shot to replicate their typical pre-shot routine. Shots were hit off artificial grass into a net, with a target positioned to provide directional focus.

Driver CHS, ball speed and smash factor were measured using a TrackMan 4 launch monitor (TrackMan A/S, Vedbæk, Denmark), operated in Indoor Mode, with participants striking Titleist ProV1 golf balls (Acushnet Company, Fairhaven, MA, USA). The device was set up according to manufacturer’s guidelines; specifically, 2.7 m behind the participant at the same height as the area the golfer was hitting from and in line with their intended target. Participants completed a minimum of three valid driver shots per session, separated by one minute of rest, striking the ball with maximal effort while maintaining their typical swing mechanics and aiming for a centred strike on the clubface. Shot validity (centredness of strike, effort, and swing characteristics) was judged from auditory, tactile and ball-flight feedback, with the investigator confirming each shot with the participant; invalid shots were discarded and repeated, up to a maximum of 10 attempts per session. Across both sessions, 24 of 28 participants needed only the minimum three trials, while four required one to three additional attempts; three valid shots per participant per session were retained for analysis (168 observations in total).

#### 2.2.2. Countermovement Jump

After receiving a standardised verbal explanation from the lead researcher, participants completed three familiarisation trials of the CMJ. All CMJs were performed on a force platform (Bertec 5060-06, Columbus, OH, USA) sampling at 1000 Hz, with the system zeroed prior to each trial. Participants were instructed to stand upright and still for the initial one second of data collection to allow for accurate determination of body weight. Upon the command ‘3, 2, 1, GO!’, participants were instructed to descend to a self-selected countermovement depth and “jump as high and as fast as possible” with hands on hips. Any trials unintentionally completed with the inclusion of an arm swing or knee flexion during the flight phase were omitted and additional trials were performed, up to a maximum of six attempts. Familiarisation trials were excluded from analysis, and three valid test trials per participant per session were retained. All valid trials were entered into the analyses, rather than the best trial or the mean, giving 168 observations for each CMJ variable. All trials were performed on a solid, even, non-slip surface and were separated by one minute of seated rest.

### 2.3. Data Analysis

Driver CHS, ball speed and smash factor were recorded for each trial, and all valid trials (168 observations) were retained for analysis. The Bertec force platform was connected directly to Hawkin Dynamics Inc. (HD Inc.) proprietary software (Westbrook, ME, USA) via the manufacturers’ integration, so raw vGRF data were processed automatically after each trial. Body weight was calculated as the lowest one-second average of vGRF during the quiet standing period, with the software requiring the standard deviation of vGRF to remain below 25 N for at least one second before movement could be initiated. Movement onset was identified when vGRF deviated from body weight by more than five standard deviations, with integration beginning from a backward search of 200 ms to the closest value of body weight [24]. Centre of mass velocity was obtained by dividing net vGRF (i.e., vGRF minus body weight) by body mass and integrating using the trapezoidal rule [25]. The propulsion phase was defined from the instant centre of mass velocity exceeded 0.01 m/s until take-off, which was identified when vGRF fell below 25 N. Jump height was calculated from take-off velocity (v) as v^2^/2 g, jump momentum as body mass multiplied by take-off velocity [26] and peak propulsive power as the maximum instantaneous product of vGRF and centre of mass velocity during the propulsion phase. All variables calculated in the HD Inc. Cloud were exported to Microsoft Excel (version 16; Microsoft Corp., Redmond, WA, USA). Definitions and calculation methods for all variables (jump height via impulse-momentum, jump momentum and peak propulsive power) are available via the HD Inc. Metric Database (www.hawkindynamics.com/hawkin-metric-database(accessed on 9 August 2026)). These variables were retained as they have been previously associated with driver CHS [12,13,14,15,16].

### 2.4. Statistical Analysis

All analyses were conducted in R (R version 4.6.1: The R Project for Statistical Computing, n.d.) using Bayesian inference, given well-documented concerns about the misinterpretation of null-hypothesis significance testing, *p*-values and confidence intervals [27].

As a joint a priori power calculation across all predictors and outcomes was not feasible, a simulation-based sensitivity check was conducted for the primary hypothesis of interest, the jump height × session interaction on CHS, using the powerbrmsINLA package [28]. A sample size of *n* = 30 was sufficient for moderate effects (0.3–0.4); following a reduction to twenty-eight participants, further simulations confirmed *n* = 28 remained sufficient for effects of 0.4–0.5. Precision for the remaining predictor–outcome combinations should be judged from their reported credible intervals rather than this sensitivity analysis.

Between-session reliability was assessed using Bayesian variance-components models (brms package [29]) fitted separately to the trial-level data for each variable, with session as a fixed effect and a random intercept for participant (i.e., variable ~ 1 + session + (1 | participant)). Weakly informative priors were used, namely normal (0, 100) for the intercept, normal (0, 10) for session and half-Cauchy (0, 10) for the participant and residual standard deviations. Models were run with four chains of 4000 iterations, including 1000 warm-up iterations (seed = 123). For each posterior draw, the intraclass correlation coefficient (ICC) was calculated as the between-participant variance divided by the total variance, and the coefficient of variation (CV) as the residual standard deviation divided by the grand mean, expressed as a percentage. Results are reported as posterior medians with 95% credible intervals (CrI). ICC values were interpreted as poor (<0.50), moderate (0.50–0.74), good (0.75–0.90) or excellent (>0.90) based on the lower bound of the 95% CrI, and CV values as excellent (<5%), good (5–10%), moderate (>10–15%) or poor (>15%) based on the upper bound.

Bayesian quantile regression models (brms package; asymmetric Laplace distribution) estimated predictor–outcome relationships at the 25th, 50th, 75th and 90th percentiles of driver CHS, ball speed and smash factor, offering a more nuanced characterisation than mean-based analyses [30]. The asymmetric Laplace likelihood was used because it allows a given conditional quantile of the outcome to be modelled directly, without assuming normally distributed residuals [31]. Separate models were fitted for each CMJ predictor, outcome and quantile. Each model included the CMJ predictor, session and a Predictor × Session interaction to quantify session-related changes in slope, alongside a random intercept for participant to account for repeated trials nested within participants and sessions (e.g., CHS ~ jump height × session + (1 | participant)). As each CMJ variable was modelled separately, the models describe how each variable relates to each outcome but were not designed to quantify the unique or incremental contribution of one CMJ variable over another. Peak propulsive power was z-score standardised, so its coefficients reflect the change per one standard deviation increase. Coefficients and 95% credible intervals (CrI) are reported alongside the posterior probability of directional effects. The 90th percentile was included to describe associations among the fastest swingers, as the upper tail is often of most practical interest in performance settings [30]. However, with 28 participants, estimates at this percentile draw on relatively few golfers and were therefore interpreted with caution. Bayesian correlations were modelled using the Bayesian First Aid package [32].

For the quantile regression models, default brms priors were used, with flat priors on population-level coefficients, a Student-t prior on the intercept and half Student-t priors on the participant-level standard deviation and residual scale, with location and scale derived from each outcome by brms. Posterior sampling used Hamiltonian Monte Carlo with the No-U-Turn sampler, with four chains of 2000 iterations, of which the first 1000 were discarded as warm-up, giving 4000 post-warm-up draws per model. Where required, adapt_delta (0.95 to 0.99) and max_treedepth (10 to 15) were increased. All models showed satisfactory convergence ($\hat{R}$ < 1.01), with minimum bulk and tail effective sample sizes ranging from 282 to 563 and from 355 to 1162, respectively, and posterior predictive checks showed good agreement between observed and simulated data.

## 3. Results

Between-session reliability data are presented in Table 1. Descriptive data for each variable by quantile are presented in Table 2. Bayesian quantile regression coefficients, representing the expected change in the outcome variable per one-unit increase in each CMJ predictor, for driver CHS and ball speed are presented in Table 3 and Table 4, respectively; coefficients for smash factor are reported in text below given their uniformly trivial magnitude. Bayesian correlations between CMJ variables and driver outcomes, modelled for direct comparison with prior correlational research in golf, were small-to-moderate for CHS (*r* = 0.27 to 0.38) and ball speed (*r* = 0.26 to 0.43) across sessions, with 95% CrI spanning zero throughout except for peak propulsive power and session-two ball speed (*r* = 0.43 [0.09, 0.72]). Correlations with smash factor were negligible and inconsistent in direction across sessions (*r* = −0.30 to 0.08).

### 3.1. Clubhead Speed

**Figure 1:**
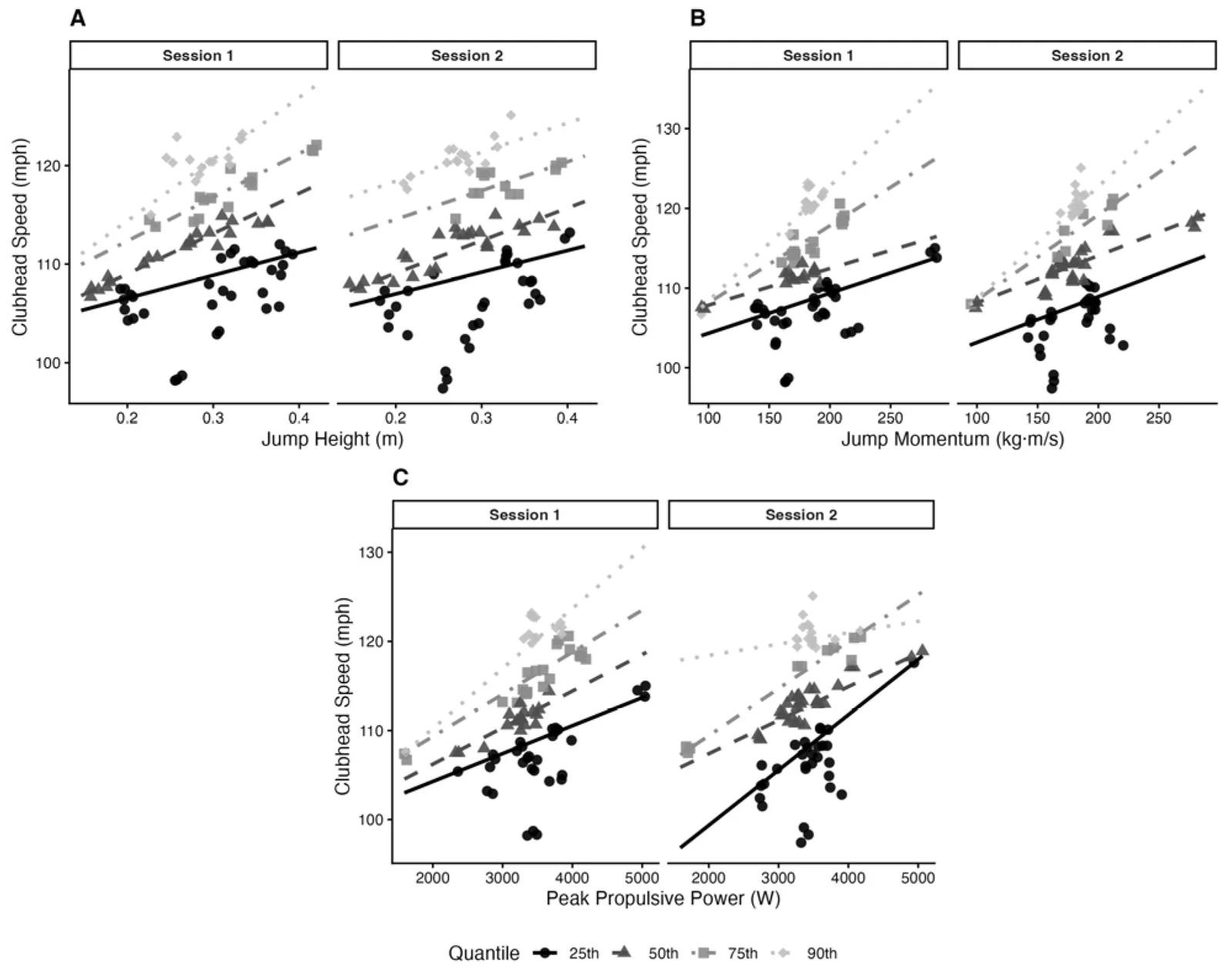
Bayesian quantile regression of driver clubhead speed (CHS) against jump height (**A**), jump momentum (**B**) and peak propulsive power (**C**) across session one and session two, showing fitted regression lines for the 25th, 50th, 75th and 90th quantiles. For visualisation only, data points are differentiated by shape according to the nearest fitted quantile line, as quantile regression does not assign individual observations to quantile groups.

Bayesian quantile regression indicated generally positive associations between all CMJ variables and driver CHS across percentiles and both sessions (Figure 1; Table 3). The most notable finding was a 98–99% posterior probability of a session-related decrease in the jump height–CHS slope at the median and upper percentiles, with a 0.05 m increase in jump height corresponding to approximately 1.3–1.4 mph in session one but a reduced effect in session two (i.e., 1.0–1.2 mph). By contrast, jump momentum and peak propulsive power demonstrated consistent positive associations across all percentiles with little evidence of session-related change. Specifically, a 10 kg·m/s increase in jump momentum corresponded to approximately 0.5–0.9 mph across the 25th to 90th percentiles (session one: 0.5–0.8 mph; session two: 0.6–0.9 mph), and a one standard deviation increase in peak propulsive power to approximately 1.7–2.0 mph (session one: 1.69–1.89 mph; session two: 1.73–2.01 mph).

### 3.2. Ball Speed

**Figure 2:**
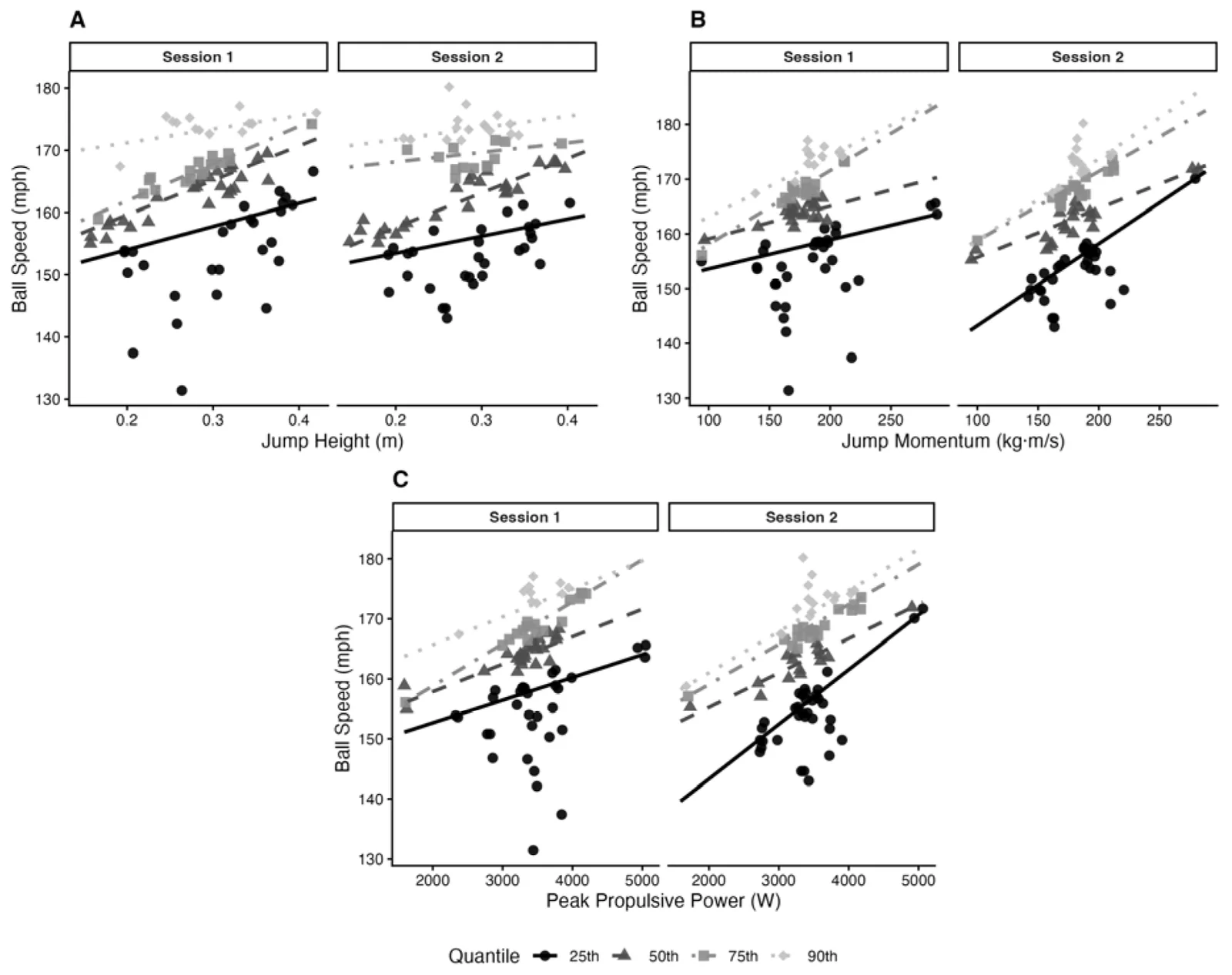
Bayesian quantile regression of driver ball speed against jump height (**A**), jump momentum (**B**) and peak propulsive power (**C**) across session one and session two, showing fitted regression lines for the 25th, 50th, 75th and 90th quantiles. For visualisation only, data points are differentiated by shape according to the nearest fitted quantile line, as quantile regression does not assign individual observations to quantile groups.

CMJ variables demonstrated uncertain associations with ball speed across all quantiles and both sessions (Figure 2; Table 4), with 95% CrI spanning zero for every coefficient. Effects were variable across the distribution; a 0.05 m increase in jump height corresponded to approximately −0.08 to 1.46 mph in session one and 0.21 to 1.62 mph in session two depending on quantile, reflecting the highly variable nature of this association across the distribution. A 10 kg·m/s increase in jump momentum corresponded to approximately −0.1 to 0.5 mph. Peak propulsive power showed the most consistent associations across session one (1.20–2.28 mph per one standard deviation increase across the 25th to 75th percentiles), and there was a probable increase in slope in session two (P(Increase) = 96–99%), although these estimates were accompanied by wide credible intervals and the 90th percentile estimate appeared anomalous. Jump momentum showed a possible increase in slope in session two (P(Increase) = 89–95%), whereas no clear session-related change was observed for jump height.

### 3.3. Smash Factor

CMJ variables demonstrated uniformly small and uncertain associations with smash factor across all quantiles and both sessions, with coefficients consistently near zero (range: −0.005 to 0.005) and credible intervals spanning zero in every case. Jump height and jump momentum showed consistently negative coefficients in session one (range: −0.001 to −0.003) that were negligible in magnitude, whilst peak propulsive power also produced small negative session one coefficients (−0.003 to −0.004) with posterior probabilities suggesting a possible increase in slope at session two (P(Increase) = 80–81%). However, all effects remained trivial regardless of quantile or session and should be interpreted cautiously given the poor between-session relative reliability of smash factor (ICC = 0.24; 95% CrI = 0.00, 0.57). Given the negligible Bayesian correlations and the uniformly trivial associations reported above, graphical or tabular representation of smash factor associations was not deemed warranted.

## 4. Discussion

To our knowledge, the present study is the first to adopt a repeated-measures Bayesian quantile regression approach to examine associations between CMJ force–time variables, CHS, ball speed and smash factor across the performance distribution in young adult male sub-elite golfers. The most important finding was that CMJ variables showed consistent positive associations with driver CHS across all quantiles and both sessions, broadly supporting earlier correlational evidence in this population [12,13,14,15]. For example, a 10 kg·m/s increase in jump momentum corresponded to a 0.5–0.9 mph increase in driver CHS, with 95% CrIs excluding zero at every quantile and slopes remaining stable between sessions. A novel finding was that the association between jump height and driver CHS was meaningfully reduced in session two at the median and upper quantiles, with a 98–99% posterior probability of a session-related decrease—a pattern not observed for jump momentum or peak propulsive power. By contrast, CMJ variables demonstrated uncertain associations with ball speed and smash factor across all quantiles and both sessions, with 95% CrIs spanning zero in almost all cases, consistent with findings from Robinson et al. [20] in elite female amateur golfers and Brennan et al. [19] in high-level amateur players. This likely reflects the role of impact efficiency factors in mediating the relationship between physical capacity and ball-flight outcomes; indeed, while lower-body force production may contribute to CHS generation, ball speed and smash factor are additionally determined by technical skill components such as club face control and strike quality that physical testing alone cannot capture [19,20].

To facilitate direct comparison with the correlational approaches employed in previous golf research, mean-based Bayesian correlations were also modelled. This comparison yielded a notably more uncertain picture; for example, jump momentum’s mean-based correlation spanned zero in both sessions, despite the quantile analyses indicating positive and well-supported associations at every percentile. This discrepancy arises because a mean-based correlation reduces the sample to a single average estimate, i.e., when the slope varies across quantiles, this average represents a compromise across heterogeneous relationships, with a corresponding loss of precision [30,33]. Reliance on mean-based correlations alone may therefore understate the strength of CMJ–CHS relationships, and the present findings reinforce recent recommendations for moving beyond mean-based analyses when characterising physical performance associations in applied sport settings [30].

The findings suggest that jump height shows positive associations with driver CHS across all quantiles in session one, with a 0.05 m increase corresponding to an approximate 1.3–1.6 mph increase in driver CHS. However, this association was likely reduced in session two at the median and upper quantiles, with the same 0.05 m increase in jump height corresponding to approximately 1.0–1.2 mph, roughly 0.2–0.3 mph less than in session one (probability of decrease = 98–99%). Jump height is independent of body mass by construction, as two athletes applying the same force per kilogram of body mass over the same time will reach the same height. Jump momentum and peak propulsive power, by contrast, scale directly with body mass [34]. As body mass is likely to be highly stable over a one-week period, any contribution of body mass to CHS would be carried into both sessions for these metrics, whereas the jump height–CHS association depends solely on how high each golfer jumped on the day. This may partly explain why the jump height–CHS slope was more sensitive to between-session variation, rather than indicating that jump height is an inferior measure. It should also be recognised that the CMJ is multidimensional, and similar jump heights can be achieved through different underlying force–time characteristics [35]. Changes in these characteristics may therefore occur between sessions without a corresponding change in jump height, which a single outcome measure cannot reveal. At the upper tail of the distribution (i.e., ≥ 90th percentile; CHS of approximately 119–122 mph), weaker and uncertain associations may additionally reflect inter-individual differences in jump mechanics such as countermovement depth, push-off distance, or limb length [36], rather than a true ceiling in physical capacity.

Jump momentum demonstrated consistent positive associations with driver CHS across all percentiles and both sessions, with a 10 kg·m/s increase corresponding to approximately 0.5–0.9 mph increase in driver CHS and little evidence of session-related changes (probability of decrease = 8–53%). However, jump momentum should be interpreted alongside jump height rather than as an independent quality. Both are derived from take-off velocity, with jump height equal to take-off velocity squared divided by twice the acceleration due to gravity, and jump momentum equal to take-off velocity multiplied by body mass [26,37]. The two therefore carry the same information about how fast each golfer left the ground, with the main difference between them being body mass. The more consistent associations observed for jump momentum may therefore partly reflect the body mass it contains, particularly as heavier golfers may also generate greater CHS [13,18]. Indeed, the association between jump momentum and sprint momentum has been shown to be inflated by the body mass common to both variables [17], and a similar coupling may apply here. From a mechanical perspective, jump momentum reflects the momentum generated during the propulsion phase [26], while body mass may also contribute to the angular momentum generated during the golf downswing, where trunk angular momentum has been identified as a primary contributor to driver CHS [38]. This is consistent with Johansen et al. [13], who reported strong associations between CMJ net impulse, mathematically equivalent to jump momentum, and driver CHS in elite male golfers (*r* = 0.78) and elite females (*r* = 0.67), and with Brennan et al. [19] who reported large to very large associations between net impulse and golf shot metrics in high-level amateur players (*r* = 0.54 to 0.62).

Peak propulsive power showed consistent positive associations with driver CHS across all quantiles and both sessions, with a one standard deviation increase corresponding to approximately 1.7–2.0 mph and little evidence of systematic session-related changes (probability of decrease = 21–44%). These findings are consistent with previous correlational evidence in golfers of varying skill levels [13,14,15] where large to very large associations between CMJ peak power and golf shot metrics have been reported (*r* = 0.63–0.65), and extend this work by characterising associations across the full performance distribution. The relationship between propulsive power and driver CHS is biomechanically plausible; power is the product of force and velocity, reflecting the ability to apply large forces at high velocities during the propulsive phase of the CMJ (201–365 ms in the present study), a time frame that closely mirrors the golf downswing (approximately 230–284 ms; [39]). While the consistent associations between propulsive power and driver CHS across sessions and the performance distribution reinforce its practical monitoring value, some caution is warranted. As already noted, power is the product of force and velocity, meaning that increases in either component will increase power, yet it is the force component, captured more directly by force–time characteristics such as propulsive force and its duration, that is most mechanistically relevant to athletic performance [40,41,42]. Moreover, the velocity term within propulsive power converges on take-off velocity, meaning that peak propulsive power carries much of the information already contained in jump height [34], while its absolute value is also heavily influenced by body mass [43]. As such, while propulsive power is a useful and accessible monitoring metric, its associations with CHS are best interpreted alongside jump height and body mass rather than as evidence of a distinct neuromuscular quality.

The comparatively weaker and more variable associations observed for ball speed and smash factor across all CMJ predictors may be partly explained by between-session relative reliability. As demonstrated in Table 1, ball speed exhibited moderate relative reliability (ICC = 0.81; 95% CrI = 0.71, 0.90), while smash factor exhibited poor relative reliability (ICC = 0.24; 95% CrI = 0.00, 0.57). Poorer relative reliability reflects less stable between-session rank ordering, which will inherently attenuate observed associations with CMJ variables regardless of the true underlying relationship [21]. In the case of smash factor specifically, poor relative reliability likely also reflects the technical homogeneity of this sub-elite sample. Golfers of this calibre tend to achieve consistently high impact efficiency, meaning smash factor scores were closely clustered with limited between-individual variation. When the range of scores on an outcome variable is restricted in this way, the magnitude of observable associations with any predictor, including CMJ variables, is inherently constrained, irrespective of any true underlying relationship [22]. This is further complicated by the fact that smash factor is a derived variable (i.e., ratio of ball speed to CHS), meaning measurement error in either component propagates through to the ratio, compounding reliability limitations [21]. Collectively, these findings retrospectively reinforce the importance of establishing between-session reliability as a prerequisite step when characterising physical performance associations in applied sport settings.

Several limitations should be acknowledged. First, the analysis was restricted to CMJ-derived variables; other physical qualities linked to driver CHS, such as isometric strength or rotational power, were not assessed [13], and future work should incorporate a broader test battery to determine each quality’s unique contribution. Second, assessments were limited to two sessions one week apart; whether CMJ–driver CHS relationships remain stable over longer periods or across competitive seasons requires longitudinal or seasonal designs. Third, the relatively small, homogeneous sample of young adult male sub-elite golfers limits generalisability to other populations, and future research should replicate these findings more broadly. Fourth, strike location on the clubface was judged from auditory, tactile and ball-flight feedback rather than measured objectively (e.g., impact tape or launch monitor impact location), which is particularly relevant to ball speed and smash factor. Similarly, beyond the instructions given between sessions, factors such as sleep, caffeine and food intake were not standardised and may have contributed to between-session variation in CMJ and driving performance. From an analytical perspective, estimates at the 90th percentile draw on relatively few golfers and should be interpreted with caution. Each CMJ variable was also modelled separately, and no body-mass-adjusted analysis was conducted, so the present data cannot show whether any one metric explains CHS beyond the others. Future work should therefore examine normalised or allometrically scaled CMJ variables, alongside force–time characteristics that describe how the jump is produced (e.g., propulsive force and duration), within a single modelling framework.

## 5. Conclusions

The present findings offer several practical implications for practitioners working with sub-elite golfers. Jump momentum and peak propulsive power showed comparatively stable associations with driver CHS across the performance distribution and both testing sessions, and may be useful complementary monitoring variables in applied golf settings. However, both scale with body mass, and their predictive value and responsiveness to training-induced changes are yet to be established. In contrast, the jump height–CHS association weakened in session two at the median and upper quantiles, suggesting that single-session jump height data should be interpreted with caution when making inferences about driving performance. As the outcome of the CMJ, and one that is independent of body mass, jump height remains a valuable measure, but it is best collected across multiple sessions and interpreted alongside body mass. Associations between CMJ variables and ball speed and smash factor were uncertain across all quantiles, reflecting the additional influence of technical factors (e.g., centredness of strike and club face control) that physical testing cannot capture. A comprehensive assessment of driving performance should therefore combine physical testing with launch monitor data. Given its poor between-session relative reliability, smash factor may also be unsuitable as a primary monitoring outcome in repeated-measures designs, whereas CHS could be prioritised as the driving outcome when tracking physical development over time, with CMJ variables interpreted alongside body mass.

## Figures and Tables

**Table 1:** Between-session reliability for variables based on Bayesian intraclass correlation coefficients (ICCs) and coefficient of variation (CV), including 95% credible intervals (CrI).

Variable	ICC ^[95% CrI]^	CV% ^[95% CrI]^
Clubhead Speed (mph)	0.98 ^[0.96, 0.99]^	0.90 ^[0.80, 1.02]^
Ball Speed (mph)	0.81 ^[0.71, 0.90]^	2.42 ^[2.15, 2.73]^
Smash Factor	0.24 ^[0.00, 0.57]^	1.72 ^[1.33, 2.21]^
Jump Height (m)	0.98 ^[0.96, 0.99]^	3.40 ^[3.03, 3.83]^
Jump Momentum (kg·m/s)	0.98 ^[0.96, 0.99]^	1.84 ^[1.64, 2.07]^
Peak Propulsive Power (W)	0.99 ^[0.99, 1.00]^	2.80 ^[2.48, 3.19]^

Abbreviations: mph, miles per hour; m, metres; kg, kilograms; s, seconds; N, Newtons; W, watts; ICC, intraclass correlation coefficient; CV, coefficient of variation; CrI, credible interval.

**Table 2:** Descriptive data for each variable with their associated means and standard deviation per quantile.

Variable	Session	Mean ± SD	0–25th	25–50th	50–75th	75–90th	90–100th
Clubhead Speed (mph)	1	112.33 ± 6.15	105.00 ± 2.95	110.00 ± 1.33	114.00 ± 1.42	119.00 ± 0.93	122.00 ± 0.90
	2	112.34 ± 6.35	104.00 ± 3.11	110.00 ± 1.36	115.00 ± 1.87	120.00 ± 0.68	122.00 ± 1.46
Ball Speed (mph)	1	162.40 ± 9.01	150.00 ± 6.74	161.00 ± 2.12	166.00 ± 1.18	171.00 ± 2.50	175.00 ± 0.99
	2	162.22 ± 8.87	151.00 ± 3.71	159.00 ± 2.21	167.00 ± 1.66	172.00 ± 0.85	175.00 ± 2.25
Smash Factor	1	1.45 ± 0.04	1.40 ± 0.03	1.44 ± 0.01	1.46 ± 0.01	1.48 ± 0.00	1.50 ± 0.03
	2	1.44 ± 0.04	1.40 ± 0.03	1.45 ± 0.01	1.46 ± 0.01	1.48 ± 0.01	1.50 ± 0.00
Jump Height (m)	1	0.29 ± 0.07	0.20 ± 0.02	0.28 ± 0.02	0.32 ± 0.01	0.35 ± 0.01	0.39 ± 0.02
	2	0.29 ± 0.06	0.20 ± 0.03	0.27 ± 0.01	0.31 ± 0.01	0.34 ± 0.01	0.33 ± 0.01
Jump Momentum (kg·m/s)	1	181.16 ± 31.94	146.00 ± 23.30	174.00 ± 5.53	188.00 ± 3.50	202.00 ± 5.65	238.00 ± 35.90
	2	180.69 ± 29.92	148.00 ± 21.90	174.00 ± 5.23	187.00 ± 3.05	199.00 ± 6.35	235.00 ± 33.50
Peak Propulsive Power (W)	1	3414.70 ± 590.03	2730.00 ± 550.00	3333.00 ± 49.20	3531.00 ± 99.20	3819.00 ± 54.50	4392.00 ± 467.00
	2	3406.71 ± 550.74	2778.00 ± 488.00	3326.00 ± 46.50	3502.00 ± 56.50	3745.00 ± 81.00	4388.00 ± 441.00

Abbreviations: mph, miles per hour; m, metres; kg, kilograms; s, seconds; W, watts.

**Table 3:** Bayesian quantile regression coefficients for the associations between countermovement jump (CMJ) variables and driver clubhead speed (CHS) at the 25th, 50th, 75th and 90th percentiles, including session effects and posterior probabilities of session-related changes in slope between session one and session two.

Variable	Quantile	Coefficient ^[95% CrI]^	Session Effect ^[95% CrI]^	P(Decrease)	P(Increase)
Jump Height (m)	0.25	32.47 ^[17.23, 46.75]^	−0.96 ^[−6.09, 4.54]^	64%	36%
	0.50	28.47 ^[16.42, 39.98]^	−4.50 ^[−8.55, −0.46]^	98%	2%
	0.75	26.09 ^[12.37, 38.23]^	−5.58 ^[−9.55, −1.52]^	99%	1%
	0.90	26.56 ^[11.42, 38.53]^	−6.46 ^[−10.76, −1.91]^	99%	1%
Jump Momentum (kg·m/s)	0.25	0.07 ^[0.03, 0.11]^	0.00 ^[−0.01, 0.01]^	53%	47%
	0.50	0.08 ^[0.04, 0.12]^	0.01 ^[−0.00, 0.02]^	8%	92%
	0.75	0.06 ^[0.02, 0.11]^	0.01 ^[−0.01, 0.02]^	15%	85%
	0.90	0.05 ^[0.01, 0.10]^	0.01 ^[−0.00, 0.03]^	8%	92%
Peak Propulsive Power (per 1 SD)	0.25	1.69 ^[0.86, 2.49]^	0.04 ^[−0.30, 0.45]^	44%	56%
	0.50	1.89 ^[1.09, 2.69]^	0.12 ^[−0.18, 0.45]^	21%	79%
	0.75	1.88 ^[0.99, 2.74]^	0.06 ^[−0.26, 0.45]^	39%	61%
	0.90	1.81 ^[0.70, 2.74]^	0.09 ^[−0.25, 0.61]^	39%	61%

Abbreviations: CrI, credible interval; m, metres; kg, kilograms; s, seconds; W, watts; P, probability. Note: Coefficients represent the expected change per one-unit increase in the CMJ variable (or per one standard deviation increase for peak propulsive power, which was z-score standardised) at each quantile. Session Effect indicates the change in that association from session one to session two. P(Decrease) and P(Increase) are complementary posterior probabilities that the session effect represents a decrease or increase, respectively.

**Table 4:** Bayesian quantile regression coefficients for the associations between countermovement jump (CMJ) variables and driver ball speed at the 25th, 50th, 75th and 90th percentiles, including session effects and posterior probabilities of session-related changes in slope between session one and session two.

Variable	Quantile	Coefficient ^[95% CrI]^	Session Effect ^[95% CrI]^	P(Decrease)	P(Increase)
Jump Height (m)	0.25	8.10 ^[−44.01, 51.82]^	2.21 ^[−14.74, 19.13]^	39%	61%
	0.50	29.20 ^[−13.58, 67.66]^	3.14 ^[−15.02, 20.01]^	36%	64%
	0.75	17.63 ^[−24.17, 57.00]^	7.08 ^[−10.87, 23.27]^	20%	80%
	0.90	−1.63 ^[−42.85, 37.31]^	5.74 ^[−12.42, 23.49]^	26%	74%
Jump Momentum (kg·m/s)	0.25	0.03 ^[−0.07, 0.12]^	0.03 ^[−0.01, 0.07]^	11%	89%
	0.50	0.05 ^[−0.04, 0.15]^	0.03 ^[−0.01, 0.06]^	7%	93%
	0.75	0.03 ^[−0.06, 0.13]^	0.03 ^[−0.01, 0.06]^	7%	93%
	0.90	−0.01 ^[−0.10, 0.09]^	0.03 ^[−0.00, 0.07]^	5%	95%
Peak Propulsive Power (per 1 SD)	0.25	1.20 ^[−1.28, 3.62]^	2.40 ^[−1.46, 6.20]^	4%	96%
	0.50	2.28 ^[−0.20, 4.60]^	3.34 ^[−0.20, 6.69]^	3%	97%
	0.75	1.86 ^[−0.57, 4.22]^	3.04 ^[0.07, 6.54]^	2%	98%
	0.90	0.0009 ^[−0.0034, 0.0052]^	0.0040 ^[0.0015, 0.0107]^	1%	99%

Abbreviations: CrI, credible interval; m, metres; kg, kilograms; s, seconds; W, watts; P, probability. Note: Coefficients represent the expected change per one-unit increase in the CMJ variable (or per one standard deviation increase for peak propulsive power, which was z-score standardised) at each quantile. Session Effect indicates the change in that association from session one to session two. P(Decrease) and P(Increase) are complementary posterior probabilities that the session effect represents a decrease or increase, respectively.

## Data Availability

The data presented in this study are available on request from the corresponding author.
